# Editorial: Intelligence and Safety for Humanoid Robots: Design, Control, and Applications

**DOI:** 10.3389/fnbot.2021.808369

**Published:** 2021-12-20

**Authors:** Zhaoquan Gu, Keke Tang, Zheng Wang, Yanhua Li, Wei Shi, Zhihong Tian

**Affiliations:** ^1^Cyberspace Institute of Advanced Technology, Guangzhou University, Guangdong, China; ^2^Department of Mechanical and Energy Engineering, Southern University of Science and Technology, Guangdong, China; ^3^Computer Science Department, Worcester Polytechnic Institute, Worcester, MA, United States; ^4^School of Information Technology, Carleton University, Ottawa, ON, Canada

**Keywords:** robotics, intelligence, safety, design, control

Humanoid robots attract growing research interests from different communities, both as tools for artificial intelligence research and neurocognitive interaction assessment, and as enabling technology with high societal impacts as personal robots for health, education, and entertainment. These robots, modeled on the basis of the embodiment of neural systems in software and hardware devices, are characterized by a high number of degrees of freedom, complex end effectors, and locomotion mechanisms on the hardware side. On the control side, they are characterized by the intrinsic and complex variety of their behavioral skills that are learned (imitation, reinforcement, statistical), for instance, learning-by-demonstration, data-driven approaches to humanoid arm programming, and the most recent AI-based approaches to manipulation and locomotion control.

Targeting at co-existing or physically interacting with humans, both intelligence and safety are of prominent importance for service-oriented humanoid robots, corresponding to the software and hardware levels, respectively. There has been recent progress in both areas, such as the quick emergence of learning-based artificial intelligence and soft robotics, bringing paradigm changes to brain-inspired humanoid design, control, and applications.

The Research Topic “Intelligence and Safety for Humanoid Robots: Design, Control, and Applications” includes 11 high-quality manuscripts that offer technological and methodological advances, as well as new features and approaches to robots.

Zhu et al. explored the challenging situation where robot grasping easily fails, and proposed a hybrid policy by combining visual cues and proprioception of the gripper for the effective failure detection and recovery in grasping. Particularly, they validated their system using a proprioceptive self-developed soft robotic gripper that is capable of contact sensing.

By investigating the fact that soft actuators usually fall short of motion accuracy and load capacity, or need large-size, bulky compressors, due to the limitations of materials and structures, Lin et al. developed a self-sensing vacuum soft actuation structure that acquires good balance among precision, output force, and actuation pressure.

Considering the huge demands for elderly-serving devices, especially for those with mobility impairment, Zhao et al. designed a novel smart robotic walker that supports multiple modes of interactions through voice, gait, or haptic touch, and allows intelligent control via learning-based methods to achieve mobility safety.

To improve the fast and stable walking ability of a humanoid robot, Tao et al. proposed a gait optimization method based on a parallel comprehensive learning particle swarm optimizer. Experimental results confirmed that the method achieved a quickly optimal solution, and the optimized humanoid robot possessed a fast and steady gait and flexible steering ability.

To enable robots to execute pre-defined tasks based on simple and direct and explicit language instructions, Li et al. developed a framework that includes a language semantics module to extract the keywords, a visual object recognition module to identify the objects, and a similarity computation algorithm to infer the intention based on the given task.

Due to the surge in demand for data, computing resources, and network infrastructure, a scalable architecture was developed by Luo et al. to optimize the image processing efficiency and response rate of the robot's vision capabilities. The implemented Rinegan extension can improve the effectiveness and efficiency of image processing.

With the high demand on robot communication, Ge et al. proposed a traffic classification framework to effectively classify encrypted network traffic using a classification network structure combining a convolution neural network and long short-term memory network capturing traffic time and space characteristics. Experimental results demonstrate that the network can classify encrypted traffic and does not require manual feature extraction.

Peng et al. investigated the challenging situation of the limited number of wild images that are available for robotics to reconstruct 3D faces, and developed an accurate geometrical consistency modeling method based on B-spline parameter domain. Experimental results demonstrate the effectiveness of their method even in a challenging scenario, e.g., limited number of images with different head poses, illuminations, and expressions.

For the agency of a public opinion early warning task, an innovative cascade virus prediction model called CasWarn was proposed by Gao et al., which can be quickly deployed in intelligent agents to effectively predict the virality of public opinion information in different industries.

To exploit the importance of relation representation learning for knowledge graphs, an encoder-decoder model, which achieves better link prediction performance was proposed by Song et al. The core procedure of it is to embed the interaction between entities and relationships, and add a gate mechanism to control the attention mechanism.

Under the trend to integrate the ideas in game theory into the research of multi-robot systems, Jin et al. proposed a team-competition model to solve a dynamic multi-robot task allocation problem. Experimental results under many different cases demonstrate the effectiveness of their method.

## Author Contributions

All authors listed have made a substantial, direct, and intellectual contribution to the work and approved it for publication.

## Funding

This work was supported in part by the National Key R&D Program of China (no. 2019YFB1706003), the National Natural Science Foundation of China (U20B2046, 61902082, and 51975268), the Guangdong Basic and Applied Basic Research Foundation (2020A1515110997), the Science and Technology Program of Guangzhou (202002030263, 202102010419, and 202102010507), the Guangdong Higher Education Innovation Group (2020KCXTD007), the Guangzhou Higher Education Innovation Group (202032854), SUSTECH-AISONO Joint Lab Grant, and the SUSTECH Education Endowment.

## Conflict of Interest

The authors declare that the research was conducted in the absence of any commercial or financial relationships that could be construed as a potential conflict of interest.

## Publisher's Note

All claims expressed in this article are solely those of the authors and do not necessarily represent those of their affiliated organizations, or those of the publisher, the editors and the reviewers. Any product that may be evaluated in this article, or claim that may be made by its manufacturer, is not guaranteed or endorsed by the publisher.

